# CAR-based immunotherapy for breast cancer: peculiarities, ongoing investigations, and future strategies

**DOI:** 10.3389/fimmu.2024.1385571

**Published:** 2024-04-12

**Authors:** Zhipu Niu, Jingyuan Wu, Qiancheng Zhao, Jinyu Zhang, Pengyu Zhang, Yiming Yang

**Affiliations:** ^1^ Clinical Medicine, China-Japan Union Hospital of Jilin University, Changchun, China; ^2^ Department of Cell Biology and Medical Genetics, College of Basic Medical Sciences, Jilin University, Changchun, China; ^3^ Department of Pharmacology, College of Basic Medical Sciences, Jilin University, Changchun, China

**Keywords:** breast cancer, CAR-T, CAR-NK, CAR-M, exosome, TME, TNBC

## Abstract

Surgery, chemotherapy, and endocrine therapy have improved the overall survival and postoperative recurrence rates of Luminal A, Luminal B, and HER2-positive breast cancers but treatment modalities for triple-negative breast cancer (TNBC) with poor prognosis remain limited. The effective application of the rapidly developing chimeric antigen receptor (CAR)-T cell therapy in hematological tumors provides new ideas for the treatment of breast cancer. Choosing suitable and specific targets is crucial for applying CAR-T therapy for breast cancer treatment. In this paper, we summarize CAR-T therapy’s effective targets and potential targets in different subtypes based on the existing research progress, especially for TNBC. CAR-based immunotherapy has resulted in advancements in the treatment of breast cancer. CAR-macrophages, CAR-NK cells, and CAR-mesenchymal stem cells (MSCs) may be more effective and safer for treating solid tumors, such as breast cancer. However, the tumor microenvironment (TME) of breast tumors and the side effects of CAR-T therapy pose challenges to CAR-based immunotherapy. CAR-T cells and CAR-NK cells-derived exosomes are advantageous in tumor therapy. Exosomes carrying CAR for breast cancer immunotherapy are of immense research value and may provide a treatment modality with good treatment effects. In this review, we provide an overview of the development and challenges of CAR-based immunotherapy in treating different subtypes of breast cancer and discuss the progress of CAR-expressing exosomes for breast cancer treatment. We elaborate on the development of CAR-T cells in TNBC therapy and the prospects of using CAR-macrophages, CAR-NK cells, and CAR-MSCs for treating breast cancer.

## 1 Introduction

Breast cancer, the most common malignancy in women, is a developmental dysplasia of malignant cells of the ductal or lobular compartment of the breast (1). According to global cancer statistics for 2020, breast cancer accounts for an estimated 11.7% of 19.3 million new cancer cases and 6.9% of 10 million cancer deaths (2). Based on the expression of estrogen receptor (ER), progesterone receptor (PR), human epidermal growth factor receptor 2 (HER-2) and Ki-67, breast cancers have been classified into Luminal A (HER2-/ER +/PR +, low proliferation), Luminal B (HER2-/ER +/PR +, high proliferation), HER2-amplified (HER2 +/ER + or PR + or ER +/PR +; HER2 +/ER-/PR-) and triple-negative breast cancer (TNBC) (HER2-/ER-/PR-) types (3). Classification according to the molecular phenotype of breast cancer guides the clinical treatment of breast cancer. Luminal A and Luminal B types are sensitive to chemotherapy and endocrine therapy (tamoxifen, fulvestrant, aromatase inhibitor) (4–6). Chemotherapy, anti-HER-2 therapy (transtuzumab), tyrosine kinase receptor inhibitors (TKIs) (lapatinib), and endocrine therapy have achieved treatment effects in HER2-positive breast cancer (7–9). However, given the lack of specific targets, endocrine therapy or HER-2 targeted therapy has a limited response in TNBC. Although chemotherapy (anthracycline plus taxane) is the main treatment modality for TNBC, problems of treatment resistance and long-term recurrence remain to be addressed (10). Improving the treatment effects and reducing the risk of TNBC recurrence are existing challenges meriting solutions.

In recent years, chimeric antigen receptor immunotherapy has emerged as a new research focus for tumor treatment (11). The first CAR-T therapy has been approved by the Food and Drug Administration (FDA) for the treatment of relapsed or refractory acute lymphoblastic leukemia (ALL), chronic lymphocytic leukemia (CLL), mantle cell lymphoma (MCL), refractory diffuse large B-cell lymphoma (DLBCL), and multiple myeloma (MM) (12). The successful application of CAR-T cell therapy to hematological tumors implies its use as a potential treatment strategy against solid tumors. Indeed, CAR-T therapy has been widely used to treat solid tumors of the respiratory, nervous system, digestive tract, urinary, breast, and female reproductive organs (13–25). The CAR-based immunotherapy has also been used in macrophages. For example, remarkable results have been achieved in the HER2-positive ovarian cancer cell line, SKOV3 (26). Interestingly, exosomes, extracellular vesicles composed of lipid bilayers released from source cells, can deliver biological molecules to target cells and exert relevant biological effects. Recent research suggests that CAR-containing exosomes show high expression of cytotoxic molecules and inhibit tumor growth, suggesting a relatively safe option compared with CAR-T therapy without acute toxicities, such as lung cancer (27, 28). Drawing on the effectiveness of CAR-therapy in tumor treatment, this review focuses on the application of CAR-based immunotherapy in breast cancer. The aim is to provide new insights and research directions for breast cancer treatment.

## 2 Application of CAR-T therapy in breast cancer

Although effective therapies are available for Luminal A, Luminal B, and HER-2 positive breast cancer, CAR-therapy has been investigated in therapeutic studies. Recent studies have reported good therapeutic results for CAR-T therapy in Luminal A type. CAR-T therapy has achieved remarkable success in treating HER-2-positive breast cancer (29). However, evidence for CAR-T therapy for Luminal B type is limited. CAR-T therapy for TNBC treatment has achieved a breakthrough (30). The following sections summarize the application of CAR-T therapy in different subtypes of breast cancer and focus on the relevant targets of TNBC.

### 2.1 Luminal A and Luminal B

Luminal A (HER2-/ER +/PR +, low proliferation) and Luminal B (HER2-/ER +/PR +, high proliferation) are breast cancer subtypes that have a better prognosis compared to other types of breast cancer (31, 32). Although endocrine therapy and chemotherapy show good therapeutic effects on Luminal A, CAR-T therapy is still under investigation to treat this subtype. For example, diganglioside GD2, a tumor-associated antigen, is expressed in a high proportion of patients with the luminal A subtype (33). Seitz et al. found that the GD2-targeted CAR-T cells exhibited good cytolytic activity against the GD2-positive Luminal A cell line MCF 7; however, no antitumor activity against the Luminal A cell line, T-47D, was demonstrated (34). Similarly, Zhang et al. found that CAR-T cells targeting MSLN could specifically kill MSLN-positive MCF 7 breast cancer cells and release secreted cytokines (35). Bajor et al. found that PD-L1 ^low^ MCF-7 cells were eliminated by PD-L1-CAR-T cells in long-term kill trials while PD-L1-CAR-T cells combined with HER-2-CAR-T cells stimulated the expression of PD-L1 on MCF-7 cells, thereby accelerating killing (36). CAR-T cells target AXL, B7-H4, EGFR, FcγRI (CD64), and HER2 and exert antitumor effects on MCF 7 and SK-BR-3 cell lines (27, 37–39). However, CAR-T treatment for Luminal B tumors is less explored. Thus, CAR-T cells for treating Luminal A and Luminal B subtypes merit investigation.

### 2.2 HER2-positive breast cancer

With overexpression of HER2 receptors driving proliferation, survival, and invasion of breast tumors, HER2-positive breast cancers (HER2 +/ER + or PR + or PR + or ER +/PR +; HER2 +/ER-/PR-) account for 15–20% of all cases (40, 41). Breast cancer with high HER2 expression has a high recurrence rate and a poor prognosis (42). Anti-HER2 monoclonal antibodies (trastuzumab and pertuzumab) exert antitumor effects in HER2-positive breast cancer by blocking HER2 signaling and activating antibody-dependent cytotoxicity (ADCC) (43, 44). However, a retrospective study of the combination of trastuzumab and pertuzumab in HER2-positive metastatic breast cancer (MBC) reported the most common adverse effects, including neutropenia (40.0%), leukopenia (34.5%), thrombocytopenia (32.7%), and diarrhea (29.1%) (45). Although TKIs show significant utility in the treatment of HER2-positive breast cancer, they can cause complications in multiple organs, especially the heart (46). Therefore, developing a treatment modality with fewer side effects and better efficacy is the research direction for HER-2-positive breast cancer.

#### 2.2.1 HER2 serves as a target site

Significant breakthroughs have been made in achieving a killing effect on HER2-positive breast tumors using CAR-T cells targeting HER2. HER2-targeted CAR-T cells specifically recognize HER2-positive tumor cells, inhibiting tumor growth *in vitro* and *in vivo (* 47). Similarly, CAR-T cells transduced with trastuzumab scFv efficiently lyse HER2-positive cancer cells, such as MCF-7 HER2 cells (genetically engineered to express HER2) and SK-BR-3 cells (27). HER2-targeted CAR-T cells and anti-PDL1 treatment together show a significant anti-tumor effect on HER2-positive breast cancer (48). However, HER2 targeting of CAR-T cells mediates off-target recognition in normal lung cells expressing HER2, in turn leading to multi-organ failure (41). Off-target toxicity of HER2-targeted CAR-T cells is challenging for achieving clinical utility of HER2-targeted CAR-T cells in breast cancer. Therefore, improving the therapeutic efficacy of HER2-targeted CAR-T cells in HER2-positive breast cancer is the research direction for treating HER2-positive breast cancer.

#### 2.2.2 Progress in CAR-T therapy for treating HER2-positive breast cancer

As CAR-T cells targeting HER2 are immature for treating HER2-positive breast cancer, several new therapeutic ideas and approaches have been proposed to improve the therapeutic effects of HER-2-targeted CAR-T cells. For example, bispecific CAR-T cells have employed to treat HER2-positive breast cancer. More importantly, the emergence of Tandem CAR-T cells (TanCAR-T) may provide new ideas and approaches for treating HER2-positive breast cancer. These studies provide new research directions for CAR-T cell treatment of HER2-positive breast cancer and improve clinical outcomes.

##### 2.2.2.1 Bispecific CAR-T cells

Bispecific CAR-T cells have shown tremendous progress in treating HER2-positive breast cancer. For instance, bispecific CAR-T cells targeting HER2 and the melanocytic protein (gp100) can eradicate various large solid tumors, including orthotopic mammary tumors expressing HER2 in the breast and brain of immunocompetent mice (49). Bispecific CAR-T cells targeting HER2 and MUC 1 showed cytotoxic activity in breast cancer (50). Thus, advancements in bispecific CAR-T cells are important for improving the therapeutic effect of HER2-targeted CAR-T cells in breast cancer.

##### 2.2.2.2 TanCAR-T cells

A cutting-edge therapeutic approach is CAR-T cells with multi-targeting capabilities, carrying more than one specific CAR, to improve the ability to target tumor cells (51). TanCAR-T cells are a novel bispecific CAR containing two scFV domain junctions, allowing a single CAR-T cell to recognize multiple tumor antigens. The two specific CARs of TanCAR-T cells can synergistically enhance the activation of T cells when recognized simultaneously. For example, tandem CAR-T targeting HER2 and IL13Rα2 enhance the antitumor effect on glioblastoma (52). TanCAR cells targeting CD19 and HER2 lyse either HER2-positive or CD19-positive target cells, accompanied by the simultaneous secretion of IFN-γ and IL-2. The two specific CARs of tandem CAR-T cells enable near-free movement of TanCAR subunits and enhanced tandem recognition (53). Thus, TanCAR cells have shown great promise for their application in treating HER2-positive breast cancer. Construction of TanCAR-T cells targeting HER2 and tumor associated antigens (TAAs), expressed highly in breast cancer may provide novel approaches to improving HER2-positive breast cancer.

### 2.3 TNBC

TNBC accounts for approximately 30% of all breast cancer deaths (54). TNBC is highly invasive and distant metastatic, with a high histological grade compared to other subtypes of breast cancer (55). The main treatment for TNBC is postoperative adjuvant chemotherapy after curative surgical resection (56). However, the high recurrence rate and poor prognosis of TNBC remain challenging. Due to the lack of clear targets, treatment of TNBC is insensitive to targeted therapy. Anthracyclines and paclitaxel are chemotherapy regimens employed for TNBC. However, extreme toxicity and side effects do not improve patient survival (57). The FDA has recently approved PARP inhibitors, PD-1 inhibitors, and an anti-trop2 antibody-drug conjugate (sacituzumab govitecan) for the combination treatment of TNBC, benefiting more patients with TNBC (58–60). However, the adverse effects of PARP inhibitors in TNBC include gastrointestinal reactions, myelosuppression, and liver function impairment (61). Only a small proportion of patients benefit from PD-L1 inhibitor monotherapy (62). The anti-trop-2 antibody-drug conjugate shows adverse effects of neutropenia (39%), leukopenia (16%), anemia (14%), and diarrhea (13%) (63). Therefore, breakthrough evidence for CAR-T in solid tumors provides new opportunities for TNBC therapy.

We summarize the effective and potential targets of CAR-T cell therapy in TNBC in **Table 1**. Among them, the effective targets refer to TAAs in TNBC, which can recognize and activate CAR-T cells expressing a single specific CAR-To produce antitumor effects. These TAAs have been verified in clinical experiments or preclinical experiments. Similarly, potential targets refer to TAAs in TNBC, which can activate CAR-NK cells expressing a single specific CAR, or TanCAR-T cells expressing multiple specific CAR. Potential targets refer to TAAs in other solid tumors that can activate CAR-T cells expressing a single specific CAR, and these TAAs are highly expressed in TNBC. Although some studies suggest the potential targets of CAR for TNBC treatment, further investigations are needed to confirm their feasibility. This review attempts to facilitate the development of new ideas for subsequent research and existing clinical applications.

**Table 1:** Summary of applications of CAR-T therapy in triple-negative breast cancer.

Category of targets	Type	Targets	Research Progress	Authors
Cell surface glycoprotein	Potential targets	CD22	Expression of CD22 on the cell membrane in TNBC cell lines (BT549 and MDA-MB-231)	Tahir Zaib et al. (64)
			CAR-T cells targeting CD22 and CD19 show significant antitumor effects in ALL	Sining Liu et al. (65)
		CD44v6	CD44v6-targeted CAR-NK cells is effective against TNBC and TME immunosuppression	Martin J. Raftery et al. (66)
		CD70	TanCAR-T cells targeting CD70 and B7-H3 exhibit significant antitumor function in the MDA-MB-435 cells	Meijia Yang et al (67)
			Targeting CD70 CAR-T cells exerts antitumor effects in AML, HNSCC, CD19-negative B cell lymphoma, and GBM	Yuk Pheel Park et al. Wenhai Deng et al. Linchun Jin et al Tim Sauerh et al Gongqiang et al (68–72)
			CD70-targeted CAR-T cell therapy for RCC has entered phase I clinical trials	Siler H Panowski et al. (73)
		EpCAM	EpCAM is overexpressed in TNBC.	Ashley Cimino et al. (74).
			Targeting EpCAM CAR-T cells exert antitumor effect in a mouse model of ovarian cancer.	Juan Fu et al. (75)
			EpCAM-specific CAR-NK-92 cells are cytotoxic to EpCAM-overexpressing colon cancer cells.	Qing Zhang et al. (76)
			The combination treatment of TY-52156 and EpCAM-targeted CAR-T cells exerts antitumor effects in EpCAM-positive 4T1 breast cancer	Ge Gao et al. (77)
		MUC1-C	CAR-T cells targeting MUC 1 exert cytotoxic effects in MUC 1-positive head and neck squamous cell carcinoma (HNSCC), hepatocellular carcinoma (HCC), intrahepatic cholangiocarcinoma (ICC), and non-small cell lung cancer (NSCLC) cells.	Zi Mei et al. Yang Chen et al. Li Mao et al. Aying Wang et al. (78–81)
			MUC1-C on the surface of TNBC can promote immune escape and progression of cancer	Takahiro Maeda et al. (82)
		FcγRI (CD64)	CD64-targeted CAR-T cells in combination with trastuzumab produce significant antitumor activity against SKBR 3 cells highly expressing HER2	Yuanbin Cui et al. (39)
		Nectin-2	Nectin-2 is a potential target for breast cancer	Tsutomu Oshima et al. (83)
			DNAM-1 CAR-NK cells can effectively recognize and kill neuroblastoma (NB) cells	Loredana Cifaldi et al. (84)
			Nutlin-3a with DNAM-1 CAR-NK cells may be a novel treatment for breast cancer	Chiara Focaccetti et al. (85)
	Effective target	αvβ3-integrin	Integrin αvβ3-targeted CAR-T cells show specific recognition and elimination of MDA-MB-231, accompanied by secretion of IFN- ɣ and IL-2	Lars Wallstabe et al. (86)
		αvβ6 integrin	Integrin αvβ6-targeted CAR-T cells co-expressing CXCR 1 or CXCR 2 can produce potent antitumor effects on the αvβ6-positive TNBC cell line, MDA-MB-468	Lynsey M. Whilding et al. (87)
		B7-H3	B7-H3-targeted CAR-T cells combined with radiotherapy improve the therapeutic efficacy on TNBC cell line, MDA-MB-231	Marco Ventin et al. (88)
		B7-H4	B7-H4-targeting CAR-T cells show cytolytic toxicity on MDA-MB-468 cells	Linlin Zhou et al. (38)
		CSPG4	CSPG4-targeted CAR-T cells show cytolytic toxicity on the TNBC cell line, MDA-MB-231	Dennis C Harrer et al. (89)
		FRα	CAR-T cells targeting FR α exert an antitumor effect on the TNBC cell line, MDA-MB-231	De-Gang Song et al. (90) Xueshuai Ye et al (91)
		FAP	FAP-targeting CAR-T cells ablate cancer-associated fibroblasts (CAFs), enhancing the antitumor activity in the TNBC cell line, HCC70	Shipra Das et al. (92)
		FcγRIII (CD16)	CD16-targeting CAR-T cells combined with cetuximab induce apoptosis in TNBC cells	Yijian Li et al (93)
		FcγRII (CD32A)	CD32A-targeting CAR-T cells in combination with cetuximab or panitumumab eliminate the MDA-MB-468 cell line overexpressing EGFR	Sara Caratelli et al. (94)
		ICAM1	ICAM1-targeting CAR-T cells effectively recognize and inhibit the growth of TNBC cells expressing ICAM 1	Heng Wei et al. (95)
		MSLN	CAR-T cells targeting MSLN can specifically kill MSLN-positive MCF 7 breast cancer cells	Qian Zhang et al. (35)
			Oncolytic adenovirus targeting TGF- β enhance the anti-tumor effects of CAR-T cells targeting MSLN for MDA-MB-231 xenografts	Yuxiang Li et al. (96)
		Nectin-4	Nectin-4-targeting CAR-T cells show a time-dependent decrease in the cellular index of the MDA-MB-453 cell line within 4 h	Fanfan Li et al. (97)
		NKG2D	NKG2D-targeting CAR-T cells exhibit antitumor activity against the NKG2DL-positive TNBC cell lines, MDA-MB-231 and MDA-MB-468	Yali Han et al. (98)
		PD-L1	CAR-T cells targeting PD-L1 exert cytotoxic effect on the TNBC cell line, MDA-MB-231	Malgorzata Bajor et al. (36)
		SSEA-4	SSEA-4-targeting CAR-T cells effectively eliminate SSEA-4-positive tumor cells	Hector J Monzo et al. (99)
			CAR-T cells targeting SSEA-4 exert antitumor effects on the MDA-MB-231 TNBC cell line showing high SSEA-4 expression.	Rita Pfeifer et al. (100)
		SLC3A2	CAR-T cells targeting SLC3A2 exert cytotoxicity in MDA-MB-231 and MDA-MB-468 cells	Giulia Pellizzari et al. (101)
		TEM8/ANTXR1	CAR-T cells targeting TEM 8 exert antitumor effects on the TNBC cell line, MDA-MB-468 and TNBC patient-derived xenografts (PDXs) models	Tiara T. Byrd et al. (102)
		TROP2	Targeting Trop2 CAR-T cells produce specific and potent cytotoxicity in the MDA-MB-231 cell line with high Trop 2 expression	Huicheng Liu et al. (103)
Receptor tyrosine kinase (RTK)	Effective target	AXL	AXL-targeted CAR-T cells exhibit antitumor activity against AXL-positive MDA-MB-231, MDA-MB-468	Zhenhui Zhao et al. (37)
		c-MET	c-Met-targeted CAR-T cells exhibit antitumor activity against the c-Met-positive TNBC cell line, BT20, and breast cancer cell line, TB129	Julia Tchou et al. (104)
		EGFR	EGFR-targeted CAR-T cells exhibit antitumor activity against MCF-7 EGFR, MDA-MB-231, and SK-BR-3 cells	Wenyan Fu et al (27)
			EGFR-targeted CAR-T cells combined with olaparib and Poly I: C inhibit the recruitment of MDSCs to enhance the efficacy against TNBC	Ruixin Sun et al. Shengmeng Di et al. (105, 106)
			EGFR-targeted T cells combined with radiotherapy effectively enhance the killing of TNBC cells	Min Zhou et al. (107)
		PTK7	PTK7-targeted CAR-T cells exert significant cytotoxic effect on PTK7-positive MDA-DB-468.	Yamin Jie et al. (108)
		VEGFR 2/3	VEGFR-2/3-targeted CART cells exert cytotoxicity against VEGFR-2 and VEGFR-3 positive breast cancer cells, accompanied by secretion of IFN-γ, TNF-α, and IL-2	Haiyan Xing et al. (109)
		ROR1	Inhibition of TGF-β receptor signaling enhance the antitumor function of ROR1-targeted CAR-T cells against TNBC	Tanja Stuber et al. (110)
			Fi-CAR-T cells targeting ROR1, which secrete anti-PD-1 scFv into the TME enhance the antitumor activity of ROR1-targeted CAR-T cells against TNBC	Micaela Harrasser et al. Chiara Corti et al. (30, 111)
	Potential targets	RON (MST1R)	MST1R is a potential new target antigen for CAR-T cell therapy in breast cancer	Wen An et al. (112)
			Overexpression of MST1R, a prognostic biomarker, is found in 50% of all human breast tumors	Brian G Hunt et al. (113)
Transmembrane-type TNF- α	Effective target	tmTNF-α	tmTNF-α-targeted CAR-T cells show efficacy against MDA-MB-231 of tmTNF-α, and the combination with PD-1 mAb increase the efficacy	Hongping Ba et al. (114)
Inactive ion channel	Effective target	nfP2X7	CAR-T cells targeting nfP2X7 show potent antitumor efficacy in xenograft mouse models of TNBC and prostate cancer	Veronika Bandara et al. (115)
ER molecular chaperones	Potential targets	csGRP78	csGRP78-targeted CAR-T cells effectively treat human pancreatic cancer in preclinical models	Yuncang Yuan et al. (116)
			Acquired tamoxifen resistance in breast cancer cells is accompanied by elevated csGRP78 levels	Chun-Chih Tseng et al. (117)
Tumor markers	Potential targets	CEA	M5A, hMN-14, and BW431/26 CAR-T cells can efficiently lyse CEA-expressing HEK293T cells and secrete IFN-γ.	Chengcheng Zhang et al. (118)
		PSMA	PSMA-targeted CAR-T cells combined with docetaxel exert a synergistic anti-tumor effect in prostate cancer	Xiaokang Zhang et al. (119)
			PSMA-targeted CAR-T cells have more potent antitumor effects in prostate cancer by blocking TGF- β signaling in T cells.	Christopher C Kloss et al. (120)
			PSMA is expressed in TNBC cells and BCSCs	Amelie Heesch et al. (121)
Tight junction proteins	Potential targets	CLDN 6	RNA vaccines can stimulate the efficacy of CLDN6-targeted CAR-T cells against solid tumors	Katharina Reinhard et al. (122)
			The ongoing phase 1/2 BNT211-01 trial has confirmed the controllable safety and efficacy of CLDN6-targeted CAR-T cells for treating solid tumors	Andreas Mackensen et al (123)
		CLDN18.2	H9 CAR-T cells can target CLDN18.2 in pancreatic tumor and melanoma	Hongtai Shi et al. (124)
			CAR-T cells targeting CLDN18.2 combined with IL-7 increase T cell infiltration in the tumor microenvironment	Hong Luo et al Hongtai Shi et al (124, 125)
			Targeting FAP CAR-T cells combined with targeting CLDN18.2 CAR-T cells exerts a stronger antitumor effect in PDAC	Yifan Liu et al. (126)
Blood coagulation factor	Potential targets	TF	TF-targeted CAR-NK cells exert a direct killing effect on TNBC cells	Zhiwei Hu (127)
Disialoganglioside	Effective target	G2D	GD2-targeted CAR-T cells can exhibit excellent cytolytic activity against GD2-positive TNBC cell line, MDA-MB-231	Christian M. Seitz et al. (34)

## 3 Effect of TME on CAR-T therapy for breast cancer

The tumor microenvironment (TME) is a dynamic cellular environment comprising innate and adaptive immune cells, stromal cells, vasculature, and acellular components, such as soluble factors, signaling molecules, and extracellular matrix (ECM) (128). TME is crucial in tumor angiogenesis, proliferation, metastasis, invasion, immune evasion, and therapeutic resistance (129). Hypoxia is a characteristic of TME. The hypoxic area is rich in MDSCs, TAMs, and Treg cells, which inhibit the activation, proliferation, and cytotoxicity of T cells, reducing the killing effect of immune response on tumor cells (130). Along with the selection of appropriate targets, the TME poses a challenge for the effective treatment of breast cancer. Targeting one or more components of the TME has research value for the treatment of malignancies. In the following sections, we present the effects of CAF, MDSCs, TAM, and Treg cells on CAR-T therapy in breast cancer treatment.

### 3.1 CAFs

CAFs are stromal cells in the breast TME, which are important for cancer progression and tumor drug resistance (131). CAFs are crucial in the TME as they participate in tumor proliferation, invasion, neoangiogenesis, inflammation, immune suppression, and the remodeling of the ECM (132). Wen et al. demonstrated that CAFs can promote tumor invasion in integrin β3-positive breast cancer cells. FAP is a therapeutic target for CAFs in the TME of HER2-positive breast cancer (133). Several studies have targeted the CAFs in the TME to improve breast cancer treatment. Rivas et al. found that targeted immunotherapy against CAFs overcame trastuzumab resistance in refractory HER2-positive breast tumors (134). In addition to treating HER2-positive breast tumors, FAP-targeted CAR-T cells can eliminate CAFs and enhance the antitumor effect in TNBC. Das et al. designed FAP-targeted CAR-T cells to ablate CAF, inhibit the recruitment of MDSC, and promote T cell infiltration, which enhanced the antitumor activity against TNBC (92). Therefore, regulating CAFs in the TME can improve the scope of CAR-T cells in breast cancer.

### 3.2 MDSCs

MDSCs, an important component of the TME, show increased levels in patients with breast cancer (135). In a xenograft mouse model constructed with TNBC cell lines, MDSCs promoted tumor progression, angiogenesis, tumor invasion, and increased metastasis (136). TR 2 expressed on MDSCs in the TME can bind TR2.41BB receptors leading to MDSC apoptosis. Therefore, treating breast cancer by targeting TR 2 has immense research potential (137). Nalawade et al. found that HER2-targeted CAR-T cells expressing the TR2.41BB costimulatory receptor exerted superior antitumor effects on HER2-positive breast cancer (138). Breast tumors recruit MDSCs to inhibit the activation and infiltration of T cells, which in turn affect the therapeutic efficacy of CAR-T cells. CAR-T cells targeting EGFR combined with olaparib or Poly I: C inhibit MDSC recruitment and enhance the efficacy of TNBC, wherein olaparib inhibit MDSCs through the SDF 1 a/CXCR 4 axis and increase the antitumor activity of CAR-T cell therapy (105, 106). Therefore, MDSCs have great research value in the application of CAR-T cells to treat breast cancer.

### 3.3 Tumor-associated macrophages

TAMs, a tumor-promoting cell type in the TME of breast cancer, include antitumor M1-like (M1-TAM) or pro-tumor M2-like (M2-TAM) TAM (139). Among them, M2-TAMs are the major components of the breast tumor stroma. TAMs are potential therapeutic targets in breast cancer. TAMs are involved in the growth, invasion, survival, angiogenesis, and metastasis of breast cancer cells (140, 141). For example, Liu et al. showed that the natural compound, emodin, blocked the epithelial mesenchymal transition (EMT) and cancer stem cell (CSC) formation in TAM-induced breast cancer cells (142). Meng et al. elucidated the molecular role of α PD-L1 in reversing TAM polarization toward the M2 phenotype in the TME of TNBC, providing novel therapeutic strategies for refractory TNBC (143). Combined treatment of FA-TLR 7-1A that transforms M2-like immune-suppressive myeloid cells into M1-like inflammatory myeloid cells with CD19-targeted CAR-T cells enhances the antitumor effects of 4T1-mCD19 breast cancer cells implanted in BALB/C mice. Therefore, CAR-T therapy has great potential for treating breast cancer by modulating TAMs.

### 3.4 Treg

Tregs are inhibitory CD4+ T cells regulated mainly by FoxP3 expression through the inhibition of T cell proliferation and secretion of inhibitory cytokines (144). Tregs are closely associated with the progression, metastasis, and local invasion of breast cancer. Núñez et al. found that Treg accumulation in patients with breast cancer was associated with breast cancer cell invasion and metastatic motility into draining lymph nodes (145). Qiu et al. demonstrated a positive correlation between CCL 5 expression levels and the degree of axillary lymph node metastasis in patients with breast cancer (146). Tregs are critical for establishing and maintaining the immunosuppressed TME to suppress antitumor immunity. Bai et al. showed that ANXA 1 enhanced the function of Treg cells and promoted the growth of breast cancer (147). Therefore, blocking ANXA 1 could reduce Treg cell function and suppress breast tumors. Small-molecule inhibitors of PDGFR β enhanced the antiproliferative activity of the anti-PD-L1 mAb against human and murine TNBC cells by reducing FOXP3 + Treg cells. Therefore, regulating the levels of Treg in the TME may be important for improving the effect of CAR-T cells in breast cancer treatment.

## 4 Toxic effects and limitations of CAR-T therapy in breast cancer

Despite the great success in tumor treatment, CAR-T therapy faces the challenges such as cytokine release syndrome (CRS), off-target effect, CAR-T cell exhaustion, and immunosuppression. Statistically, almost two-thirds of patients treated with CAR-T develop CRS, resulting in a cytokine storm, organ failure, and even death (148). When CAR-T cells were cocultured with 4T1-EGFR cells *in vitro*, they produce IL-2, IFN- γ, TNF- α, and granzyme B, which is a sign of CRS (107). CAR-T cells generated from Th/Tc17 cells combined with STING agonists or cGAMP greatly enhanced the antitumor effects on breast cancer *in vivo*, however, treatment efficacy correlated with the development of CRS (149). Although CAR-T treatment specifically kills tumor cells expressing target antigen, unexpectedly, CAR-T cells injected into the body kill normal cells expressing the target antigen, causing serious side effects (150). The first clinical trial using HER2-targeted CAR-T cells for the treatment of metastatic HER2-positive breast cancer (NCT00924287) was terminated due to organ failure of normal HER2-expressing lung tissue by off-target recognition (151). In addition, maintaining the CAR persistence force of T cells *in vivo* remains challenging. Due to continuous antigen stimulation, the expression of inhibitory receptors of the CAR construct was increased, which cause CAR-T cell exhaustion resulting in a dysfunctional state in the absence of antigen-specific T cells (152). Excitingly, the latest research developed an endogenous signaling molecule activating (ESMA) CARs which triggers robust cytotoxic activity and proliferation of the T cells when directed against the TNBC cell line MDA-MB-231 while exhibiting reduced cytokine secretion and exhaustion marker expression (153). This novel CAR design may provide a new approach to overcome T cell exhaustion. Thus, there are still several challenges to CAR-T therapy in treating breast cancer. To improve the role of CAR-T therapy in breast cancer treatment and reduce the toxic side effects of CAR-T therapy, identifying specific targets and developing persistent CAR-T cells are necessary. Accordingly, some studies have broken the innovative idea based on the existing CAR-T cell therapy, choosing to express CAR-through gene editing in other cells, such as NK cells, macrophages, and MSC surface. This breakthrough has yielded great results.

## 5 Modern advancements in CAR-based immunotherapy in breast cancer

**Figure 1:**
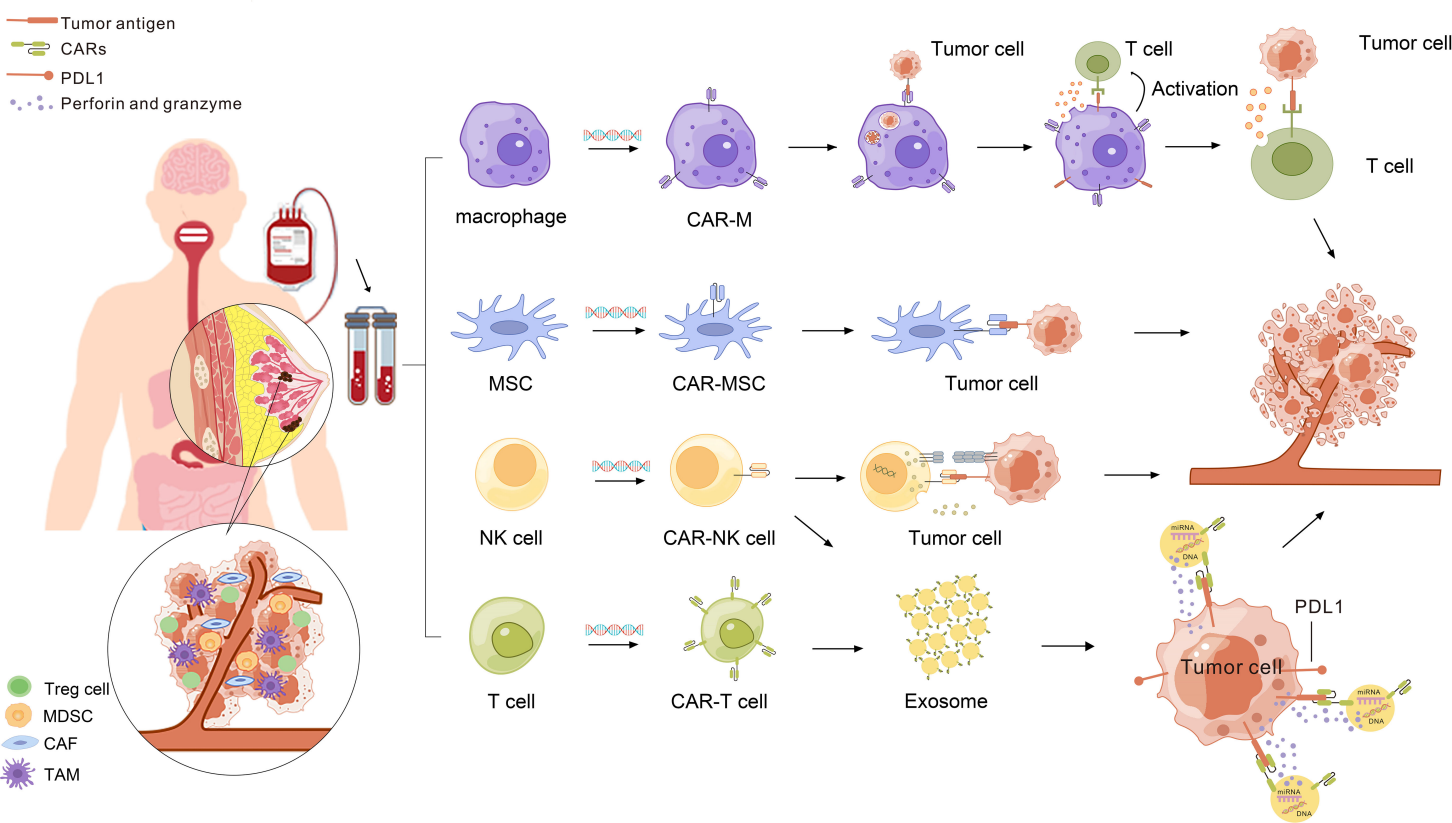
Modern advancements in CAR-Therapy for breast cancer.

In addition to CAR-T therapy, NK cells, macrophages, and mesenchymal stem cells (MSCs) can be modified using CAR-To serve as therapeutic tools for tumors (**Figure 1**). In the treatment of hematological tumors, specifically targeted CAR macrophages (CAR-Ms) can produce superior antitumor effects against CD19-positive ALL cancer cells and HER2-positive human chronic myeloid leukemia passage cells (154). Specific CAR-macrophages (CAR-Ms) can exert antitumor effects in solid tumors, such as in the HER2-positive ovarian cancer cell line, SKOV3, GD2-expressing neuroblastoma, and GD2-expressing melanomas (155–157).Thus, CAR-NK cells, CAR-M, and CAR-MSCs are beneficial for breast cancer treatment. We list effective targets in CAR-based immunotherapy for breast cancer in **Table 2** and summarize the progress.

**Table 2:** Summary of advancements in CAR-based immunotherapy for breast cancer.

Classification	Type	Target	Research progress	Author
CAR-NK	Cell surface glycoprotein	CD44v6	Anti-tumor effect on TNBC	Martin J Raftery et al. (66)
		B7-H6	Induction of death of fulvestrant-resistant breast cancer cells	You-Zhe Lin et al. (158)
		PD-L1	Treatment of TNBC by killing MDSCs	Kellsye P Fabian et al. (159)
	Receptor tyrosine kinase (RTK)	EGFR	Inhibition of brain metastases in breast cancer	Xilin Chen et al. (160)
		HER2	Stronger antitumor effects on HER2-expressing breast cancer cell lines, BT-474, SKBR 3, and MDA-MB453	Ana L Portillo (161)
			Coexpression with sPD-1 shows greater cytotoxicity against breast cancer cells with high HER2 and PD-L1 expression	Wenjiao Xia et al. (162)
	blood coagulation factor	TF	Antitumor effects in TNBC	Zhiwei Hu (127)
CAR-M	Cell surface glycoprotein	MSLN	Clinical experiments are underway	Giuseppe Schepisi et al. (163)
	Receptor tyrosine kinase (RTK)	VEGFR2	Activation by TLR 4 or IFN-γ receptor can inhibit breast cancer growth	Zhaojun Duan et al. (164)

### 5.1 CAR-Ms

Based on the specificity of CAR, CAR-M results in antigen-specific phagocytosis and tumor clearance (26, 165). CAR-Ms express pro-inflammatory cytokines and chemokines, resulting in the transition of M2 macrophages into a pro-inflammatory (M1) phenotype, induction of a pro-inflammatory TME, and resistance to the effects of immunosuppressive cytokines (155). CAR-Ms have shown advancements in the treatment of breast cancer. Duan et al. found that VEGFR-targeted CAR macrophages activated by TLR 4 or IFN- γ receptors exerted antitumor effects in 4T1 breast cancer-bearing mice (164). Clinical experiments on CAR-M targeting MSLN for breast cancer are underway and may provide new methods for the treatment of breast cancer using CAR-M (163).

### 5.2 CAR-NK cells

Genetically engineered NK cells can express CAR. These CAR-NK cells can specifically recognize and kill tumor cells (166). Similar to CAR-T cells, the CAR structure of CAR-NK cells comprises an extracellular antigen-binding region, transmembrane domain, and intracellular activation domain (167). Due to the difference in the extracellular domain, CAR-NK cells can identify different targets to treat breast cancer. CAR-NK cells targeting CD44v6, HER2, TF, B7-H6, EGFR, and PD-L1 have been successfully used for treating different types of breast cancer (66, 127, 158, 161, 162). Raftery et al. demonstrated the significant anti-tumor effect of CD44v6-targeted CAR-NK cells in TNBC (66). Hu confirmed the ability of TF as a novel target for CAR-NK cell immunotherapy in TNBC. TF-targeted antibody-like immunoconjugate (L-ICON) enhanced its efficacy *in vitro* (127). Lin et al. found that targeting B7-H6 CAR-NK cells induced the death of fulvestrant-resistant breast cancer cells (158). CAR-NK cells can be used to treat HER2-positive breast cancer. CAR-NK cells targeting HER2 produced stronger antitumor effects on the HER2-expressing breast cancer cell lines, BT-474, SKBR 3, and MDA-MB453 (161). Compared with the HER2-targeted CAR-NK cells, Xia et al. found that HER2-targeted CAR-NK cells coexpressing sPD-1 exerted greater cytotoxicity against HER2-positive breast cancer cells with high HER2 and PD-L1 expression (162).

HER2-targeted CAR-NK cells showed stronger anti-tumor effects in HER2-positive breast cancer compared with HER2-targeted CAR-T cells. HER2-targeted CAR-NK cells maintained high cytotoxic functions in the presence of immunosuppressive factors enriched in the solid tumor TME. HER2-targeted CAR-NK cells did not cause off-target toxicity to human lung epithelial cells physically expressing HER2, which may predict the potential superiority of HER2-targeted CAR-NK cells in treating breast cancer (161).

The TME can affect the antitumor function of CAR-NK cells (168). CAR-NK cells target the matrix components to remodel the TME. Fabian et al. demonstrated that NK cells targeting PD-L1 cured TNBC by killing MDSCs (159). CAR-NK cells show good curative effect in treating distant metastatic lesions of breast cancer. Chen et al. demonstrated that CAR-NK cells targeting EGFR treated brain metastasis in breast cancer (160). Thus, the progress of CAR-NK cells in breast cancer treatment provides new ideas and methods for its clinical treatment.

### 5.3 CAR-MSCs

MSCs are involved in immune response, secrete cytokines, and enhance healing (169). MSCs target different antigens by genetically engineering expression-specific CAR, providing new ideas and approaches for cancer treatment. Golinelli et al. found that GD2-targeted CAR-MSC expressing TRAIL exerted antitumor effects on GD2-positive Ewing sarcoma (ES) and glioblastoma (GBM) (170, 171). Although studies on CAR-MSC therapy are scarce, its great potential in the treatment of breast cancer has been suggested.

## 6 Clinical studies on CAR-based immunotherapy for breast cancer

Ongoing clinical studies on CAR-based immunotherapy in breast cancer are presented in the table below. These include only one CAR-NK cell and one CAR-M cell therapy for breast cancer, and most of them are CAR-T cell therapies in the clinical studies of breast cancer treatment. With the development of clinical research, we believe that CAR-based immunotherapy can provide a reference for subsequent experimental research and contribute to the development of clinical treatment. Most clinical experiments are studies on the treatment of TNBC and HER2-positive breast cancer. Among them, only one is a study on CAR-M, and one is on CAR-NK cell therapy for breast cancer. Clinical experiments with CAR-TILs are underway, which may provide new perspectives for the treatment of breast cancer. With the development of clinical experiments, the application of CAR-based immunotherapy in breast cancer treatment can provide us with new experimental evidence and reference. We used (https://clinicaltrials.gov/search? Cond = Breast% 20 Cancer & amp; intr = CAR-T) for exploring clinical trials for breast cancer, and **Table 3** provides a reference.

**Table 3:** Ongoing clinical studies on CAR-based immunotherapy for breast cancer.

NCT Number	Study Status	Conditions	Interventions	Phases	Enrollment	Completion Date
NCT05686720	NOT_YET_RECRUITING	Advanced TNBC	SZ011 CAR-NK cell	EARLY PHASE1	12	2024/6/1
NCT05341492	RECRUITING	EGFR/B7H3-positive advanced TNBC	EGFR/B7H3-targeted CAR-T cell	EARLY PHASE1	30	2025/5/1
NCT05274451	RECRUITING	TNBC	LYL797(ROR1-targeted CAR-T cell Therapy)	PHASE1	54	2026/9/1
NCT05239143	RECRUITING	Breast Cancer	MUC1C-targeted CAR-T cells	PHASE1	100	2039/4/1
NCT04842812	RECRUITING	Breast Cancer	CAR-TILs	PHASE1	40	2035/1/1
NCT04660929	RECRUITING	HER2-positive Breast Cancer	CT-0508(HER2-targeted CAR macrophages)	PHASE1	48	2024/12/1
NCT04511871	RECRUITING	Breast Cancer	CCT303-406 CAR modified autologous T cells	PHASE1	15	2025/3/29
NCT03740256	RECRUITING	Breast Cancer	CAdVEC, HER2 specific CAR-T cells	PHASE1	45	2038/12/30
NCT03696030	RECRUITING	HER2-positive Breast Cancer	HER2-targeted CAR-T cell	PHASE1	39	2025/2/28
NCT02839954	UNKNOWN	TNBC	Anti-MUC1 CAR-pNK cells	PHASE1|PHASE2	10	2018/7/1
NCT01837602	COMPLETED	Metastatic Breast Cancer, TNBC	cMet-targeted CAR-T cells	PHASE1	6	2018/8/13
NCT05812326	COMPLETED	Advanced Breast Cancer, Breast Neoplasm Malignant Female	DRUG: AJMUC1- PD-1 gene knockout anti-MUC1 CAR-T cells	PHASE1|PHASE2	15	2022/11/16

## 7 Therapeutic applications of CAR exosomes in breast cancer

Exosomes, comprising lipid bilayers, can transfer miRNA, mRNA, and proteins from the original cells to the corresponding target cells (172). Exosomes secreted by cells can be transmitted to target cells through the humor, causing changes in the physiological functions of target cells (173). Similarly, exosomes are crucial in tumor cell invasion and metastasis (174). Based on the disease-treating properties of exosomes, CAR-based cell-derived exosomes may have great potential for breast cancer (175). Interestingly, exosomal expression shows the same level of CAR as the protocells (27). Based on this feature, CAR-T cell-derived exosomes and CAR-NK cell-derived exosomes exerted antitumor effects in breast cancer cell lines (**Table 4**). Therefore, exosomes carrying CAR may provide new therapeutic means for breast cancer treatment. Exosomes carrying CAR offer hope for patients with breast cancer and may provide solutions to overcome the challenge of expense of CAR-T therapy and its wide accessibility (**Figure 1**).

**Table 4:** Summary of advancements in research on CAR-T cell-derived exosomes and CAR-NK cell-derived exosomes for breast cancer.

Classification	Target	Subtypes	Research progress	Author
CAR-T cell-derived exosomes	EGFR	TNBC	Transduction with cetuzumab shows dose-dependent TGI in the MDA-MB-231 mouse xenograft model.	Wenyan Fu et al. (27)
	HER2	HER-2-positive	Exosomes produces from trastuzumab-transduced CAR-T cells show significant antitumor effects on the treatment of MCF-7 HER2 cells and SK-BR-3 cells.	Wenyan Fu et al. (27)
	MSLN	TNBC	Exosomes derived from MSLN-targeted CAR-T cells show antitumor effects on MSLN-positive TNBC	Pengxiang Yang et al. (176)
CAR-NK cell-derived exosomes	HER2	HER2-positive	CAR-NK cell-derived exosomes and transferrin receptor binding peptide (T7) constitute a nanoplatform capable of crossing the blood-brain barrier. This nanoplatform selectively exerts antitumor effects on HER2-positive breast cancer cells in the brain through CAR on the exosomal surface.	Bolong Tao et al. (177)

Production and killing processes of CAR-M, CAR-NK, CAR-MSC, CAR-T cell-derived exosomes, and CAR-NK cell-derived exosomes. Macrophages are genetically engineered to express specific CARs. After CAR-M recognizes and phagocytoses tumor cells expressing a specific antigen, CAR-M cells present and activate T cells. Activated T cells exert anti-tumor effects. NK cells, T cells, and MSCs have been genetically engineered to express CAR. CAR-NK cells and CAR-MSC recognize and kill tumor cells. CAR-NK cells and CAR-T cell-derived exosomes can exert killing effects on tumor cells and are not inhibited by PDL 1.

### 7.1 CAR-T cell-derived exosomes in breast cancer

CAR expression has been investigated in CAR-T cell-derived exosomes. CAR-T cell-derived exosomes carry the same CAR as CAR-T cells, at comparable levels (27). Antigen-stimulated CAR-T cells show higher CAR content than exosomes stimulated with CD3/CD28 beads (27).With the deepening of research, the application scheme of CAR-T cell exosomes is gradually maturing. Peripheral blood samples from patients with cancer have been separated by density and co-cultured with CD3 and CD28 antibodies. Subsequently, CAR-T cells have been prepared using viral or non-viral transfection techniques and expanded *in vitro* in the presence of IL-2. CAR-T cell-derived exosomes have been isolated from the culture medium (178). CAR-T cell-derived exosomes have strong and specific cytotoxic activity against cancer cells. FasL, Apo 2 L, perforin, granzyme A and B play important roles (179).With the CAR and cytotoxic activities of exosomes, CAR-T cell-derived exosomes can be used to treat tumors. In the treatment of hematological tumors, targeting CD19 CAR-T cell-derived exosomes induce mRNA expression of the pro-apoptotic genes, BAD, BAX, and caspase-3 in leukemic B cells without the accompanying signs of cytotoxicity (180). CAR-T cell-derived exosomes can deliver biologically small molecules to the target cells, leading to changes in their physiological functions. For example, RN7SL1 is an RNA that can activate the RIG-I/MDA 5 signaling pathway to promote CAR-T cell expansion and effector memory differentiation (181). RN7SL1 improves the immunostimulatory properties of myeloid and DC subsets through the transfer of CAR-T cell-derived exosomes to MDSC (182). CAR-T cell-derived exosomes are less studied in drug transport. Therefore, the use of CAR-T-derived exosomes for transport drugs has great potential in the field of tumor therapy.

CAR exosomes have made tremendous progress in breast cancer treatment. With its specific CAR, CAR-T cell-derived exosomes transduced with cetuximab or trastuzumab demonstrated significant antitumor activity. Among them, exosomes expressing cetuximab scfv exerted dose-dependent tumor growth inhibition (TGI) against an EGFR-positive MDA-MB-231 mouse xenograft model. In contrast, those expressing trastuzumab scfv showed significant antitumor effects on HER-2-positive MCF-7 cells and SK-BR-3 cells in breast cancer tumor growth inhibition (TGI) (183). Yang et al. found that exosomes derived from MSLN-targeted CAR-T cells retained CAR-targeting MSLN. Exosomes carrying this CAR exerted antitumor effects on MSLN-positive TNBC without significant side effects. Retaining MSLN-targeted CAR, exosomes derived from MSLN-targeted CAR-T cells exerted significant antitumor effects on MSLN-positive TNBC without any significant side effects (176).

CAR-T cell-derived exosomes show several significant advantages. First, PD-L1 inhibited the antitumor immunity of CAR-T cells but did not affect exosomes. No CRS and off-tumor responses were observed after CAR exosomal immunotherapy. Exosomes can cross the blood-brain barrier, which may provide new therapeutic approaches for the treatment of intracranial metastases (27). CAR-T cell-derived exosomes are superior for solid tumors compared with CAR-T cells (184). In conclusion, CAR-T cell-derived exosomes possess specific cytotoxic activity against breast cancer cells and have great research potential in the development of novel breast cancer therapies.

### 7.2 CAR-NK cell-derived exosomes for the treatment of breast cancer

In addition to CAR-T cell-derived exosomes, CAR-NK cell-derived exosomes, an important component of immune cell function, carry several perforins and granzymes for the treatment of malignant tumors (185). CAR-NK are off-the-shelf cell therapies for solid tumors. For example, brain metastasis of HER2-positive breast cancer (HER2-positive BCBM) is a refractory malignancy with a high recurrence rate and poor prognosis. Interestingly, the CAR-NK cell-derived exosomes and transferrin receptor binding peptide (T7) can constitute a nanoplatform capable of crossing the blood-brain barrier. This nanoplatform selectively exerts antitumor effects on HER2-positive breast cancer cells in the brain through CAR on the surface of the exosomes (177). Studies on CAR-NK cell-derived exosomes are scarce, and there is great potential to investigate the therapeutic application of CAR-NK cell-derived exosomes in breast cancer.

## 8 Discussion

As the most common malignant tumor in women, the high incidence and mortality rate of breast cancer remains a pressing issue. With the increase in the number of clinical studies, although CAR-T therapy has achieved good therapeutic results in lumen type A, its therapeutic application in lumen B has not been reported. No CAR-T therapy for Luminal A and Luminal B has reached the clinical study stage, and huge gaps need to be supplemented. As for the therapeutic application of CAR-T cell therapy in HER2-positive breast cancer, although HER2-targeted CAR can effectively produce an antitumor effect on HER-2-positive breast cancer, the off-target effects in clinical research remains a difficult problem. Despite studies on immunomodulators, BsAbs, TanCAR, bi-specific CAR-T cells, and TME to improve the therapeutic status of HER2-targeted CAR-T cells, a recognized effective approach to improve HER2-targeted CAR-T cells for HER-2-positive breast cancer is lacking. For TNBC treatment, new effective targets of CAR-T cells are constantly being investigated. New potential targets await experimental confirmation. Clinical experiments of CAR-T cells for breast cancer are few and there is massive scope for development. We believe that with the increase of animal experiments and clinical experiments, CAR-T cell therapy with higher efficacy and fewer side effects will be applied to breast cancer treatment.

In addition to the booming development of CAR-T cell therapy in cancer treatment, research on CAR-NK cells, CAR-M, and CAR-MSC has made great progress in recent years. Although CAR-NK cells targeting CD44v6, HER2, TF, B7-H6, EGFR, and PD-L1 have made great progress in breast cancer, developing targets for CAR-NK cells in breast cancer has great research potential. CAR-Ms targeting VEGFR and MSLN have been investigated for breast cancer treatment, and suitable targets await further investigation. CAR-MSCs can be used to treat solid tumor therapy, which may provide new avenues for breast cancer treatment and merit further investigation. Although there are a few clinical studies on CAR-NK cells and CAR-Ms, their clinical effects await validation. Moreover, there are no studies of CAR-MSC for breast cancer and there is immense potential.

CAR-based immunotherapy faces great challenges in breast cancer treatment. On the one hand, the common toxic effects and limitations of CAR-T therapy hinder its application, and the development of more specific CAR-T cells remains a challenge. On the other hand, although CAR-NK and CAR-Ms have achieved more significant therapeutic effects in breast cancer treatment compared to CAR-T cells, clinical studies on their side effects are scarce. Therefore, the side effects of CAR-based immunotherapy merit further investigations to ensure that this treatment is effective and safe before entering the clinical stage.

In addition to studying specific targets to kill breast cancer cells, the TME of breast cancer is equally important in CAR-based immunotherapy and can serve as an adjuvant. Although CAFs, MDSCs, TAMs and Tregs in the TME exert a therapeutic effect, evidence remains scarce, especially for Tregs and TAMs. TME regulation can produce a synergistic effect on CAR-based immunotherapy and enhance the anti-tumor effect.

Interestingly, CAR-T cell-derived exosomes and CAR-NK cell-derived exosomes have been successfully used for breast cancer treatment, and show many advantages besides good anti-tumor effects. However, there are few studies on CAR-T cell-derived exosomes and CAR-NK cell-derived exosomes for breast cancer and no clinical studies. Exosomes expressing CAR can be used as an effective and extensive tool for breast cancer treatment.

In conclusion, we firmly believe that the present use of the target development and clinical research of CAR-based immunotherapy in breast cancer therapy could benefit a larger number of patients. The emergence of CAR exosomes may provide a more efficient, widely applicable, and low-cost tool for breast cancer treatment. In the future, CAR-based immunotherapy for breast cancer treatment is expected to benefit more patients.

## Author contributions

ZN: Writing – original draft, Conceptualization. JW: Writing – original draft. QZ: Writing – original draft. PZ: Writing – original draft. JZ: Writing – review and editing, Data curation. YY: Writing – review & editing, Conceptualization, Funding acquisition, Supervision.
